# Meta-Analysis of *Grainyhead-Like* Dependent Transcriptional Networks: A Roadmap for Identifying Novel Conserved Genetic Pathways

**DOI:** 10.3390/genes10110876

**Published:** 2019-10-31

**Authors:** Nishanthi Mathiyalagan, Lee B. Miles, Peter J. Anderson, Tomasz Wilanowski, Brian L. Grills, Stuart J. McDonald, M. Cristina Keightley, Agata Charzynska, Michal Dabrowski, Sebastian Dworkin

**Affiliations:** 1 Department of Physiology, Anatomy and Microbiology, La Trobe University, Bundoora 3086, VIC, Australia; 19551056@students.latrobe.edu.au (N.M.); lee.miles@monash.edu (L.B.M.); b.grills@latrobe.edu.au (B.L.G.); 2 School of Biological Sciences, Monash University, Clayton 3168, VIC, Australia; 3 Australian Craniofacial Unit, Women and Children’s Hospital, Adelaide 5005, SA, Australia; haemro2@hotmail.com; 4 Faculty of Health Sciences, University of Adelaide, Adelaide 5005, SA, Australia; 5 Nanjing Medical University, Nanjing 210029, China; 6 Institute of Genetics and Biotechnology, Faculty of Biology, University of Warsaw, Pawinskiego 5a, 02-106 Warsaw, Poland; t.wilanowski@biol.uw.edu.pl; 7 Department of Neuroscience, Central Clinical School, Monash University, 99 Commercial Rd., Melbourne 3004, VIC, Australia; stuart.mcdonald@monash.edu; 8 Department of Pharmacy and Biomedical Sciences, La Trobe University, Bendigo 3552, VIC, Australia; c.keightley@latrobe.edu.au; 9 Nencki Institute of Experimental Biology, Polish Academy of Sciences, 3 Pasteur St.02-093 Warsaw, Poland; a.charzynska@nencki.gov.pl (A.C.); m.dabrowski@nencki.gov.pl (M.D.)

**Keywords:** Grainyhead, Grhl, epithelia, cleft palate, craniofacial, meta-analysis

## Abstract

The *Drosophila grainyhead* (*grh*) and vertebrate *Grainyhead*-*like* (*Grhl*) transcription factors are among the most critical genes for epithelial development, maintenance and homeostasis, and are remarkably well conserved from fungi to humans. Mutations affecting *grh/Grhl* function lead to a myriad of developmental and adult onset epithelial disease, such as aberrant skin barrier formation, facial/palatal clefting, impaired neural tube closure, age-related hearing loss, ectodermal dysplasia, and importantly, cancers of epithelial origin. Recently, mutations in the family member *GRHL3* have been shown to lead to both syndromic and non-syndromic facial and palatal clefting in humans, particularly the genetic disorder Van Der Woude Syndrome (VWS), as well as spina bifida, whereas mutations in mammalian *Grhl2* lead to exencephaly and facial clefting. As transcription factors, Grhl proteins bind to and activate (or repress) a substantial number of target genes that regulate and drive a cascade of transcriptional networks. A multitude of large-scale datasets have been generated to explore the *grh*/*Grhl*-dependent transcriptome, following ablation or mis-regulation of *grh*/*Grhl*-function. Here, we have performed a meta-analysis of all 41 currently published *grh* and *Grhl* RNA-SEQ, and microarray datasets, in order to identify and characterise the transcriptional networks controlled by *grh*/*Grhl* genes across disparate biological contexts. Moreover, we have also cross-referenced our results with published ChIP and ChIP-SEQ datasets, in order to determine which of the critical effector genes are likely to be direct *grh/Grhl* targets, based on genomic occupancy by *grh*/*Grhl* genes. Lastly, to interrogate the predictive strength of our approach, we experimentally validated the expression of the top 10 candidate *grhl* target genes in epithelial development, in a zebrafish model lacking *grhl3*, and found that orthologues of seven of these (*cldn23, ppl, prom2, ocln, slc6a19*, *aldh1a3*, and *sod3*) were significantly down-regulated at 48 hours post-fertilisation. Therefore, our study provides a strong predictive resource for the identification of putative *grh*/*grhl* effector target genes.

## 1. Introduction

Among the earliest tissues formed during embryonic development are epithelia, the thin, cellular layers that line the surfaces of all organs and body cavities. More than just semi-permeable “barriers”, epithelia are, in fact, highly dynamic tissues, actively regulating embryonic growth and expansion, as well as providing essential biochemical and mechanical signal-transduction cues. Understanding the nature of the genetic networks that regulate normal epithelial development is therefore a cornerstone of understanding birth defects due to epithelial aberrations. These not only include significant congenital abnormalities, (e.g., neural tube defects, cleft lip/palate, intestinal dysfunction), but, importantly, also underpin numerous epithelial disorders in later life (e.g., psoriasis, eczema, ectodermal dysplasia and, critically, cancers). Therefore, identifying the genes that drive epithelial formation and maintenance is a necessary first step to developing therapies that may ultimately ameliorate both the severity and incidence of congenital and adult onset epithelial pathologies.

Among the most critical genes for epithelial development and homeostasis are the highly conserved *Grainyhead-like* (*Grhl*) transcription factors. First described in *Drosophila* [1] as *grainyhead* (*grh*), this gene family is found in all organisms within the animal kingdom, and is remarkable both for its extremely high evolutionary conservation across eukaryotes [2,3], and for the extensive and disparate functions that are controlled by its various orthologues, in the context of both embryonic development and adult onset disease. In *Drosophila*, *grh* regulates key processes of epithelial development, such as Planar Cell Polarity (PCP), formation of the chitinous head “skeleton”, dorsal hole closure, and cuticle integrity and maintenance [1], whereas fungal *Grhl* is essential for cell wall remodelling and asexual spore (conidial) separation [4]. In vertebrates, *Grainyhead-like* orthologues display a conserved functional homology of regulating processes, required for epithelial development. Studies in mice [2,5], *Xenopus* [6] and zebrafish [7] have determined remarkable conserved functional homology to *Drosophila grh*, apparent in the morphogenic development of the epidermal barrier [8,9,10,11], closure of the neural tube [12,13,14,15], maintenance of neural cell survival [16], and the growth and development of the craniofacial skeleton [17,18]. Together, these findings point to a critical role for *Grhl* function in the development of all organisms within the animal kingdom.

Recent work has also shown that *Grhl* function is highly conserved in humans, and that these genes regulate both development and adult disease. Critical recent work has demonstrated that loss of *GRHL3* leads to the craniofacial defect Van der Woude Syndrome (VWS), characterised by clefting of the secondary palate. This anomaly is due to aberrant maintenance of the oral periderm, a thin, extra-epithelial tissue that lines the developing palatal shelves [19]. Additionally, *GRHL3* mutations are also associated with non-syndromic palatal clefting [20] and spina bifida [21]. As substantial, disparate craniofacial defects are also observed in *Drosophila*, zebrafish and murine models lacking *grh/Grhl2/Grhl3* function, and, as these factors are expressed almost exclusively in epithelia, it becomes clear that both clefting and other facial, jaw and skull deformities observed in these models are secondary to a primary epithelial defect, following abrogation of *grh/Grhl* function.

After development, *Grainyhead-like* (*Grhl*) plays an important a role in regulating genes required for the maintenance of the epithelial barrier during growth and injury. Studies of human *Grainyhead-like* (*Grhl*) orthologues have begun to uncover the role that loss of function or gene variants have in adult-onset diseases, such as hearing loss/deafness [22,23], defects of epidermal integrity and, particularly, numerous forms of cancer [9,24,25,26]. The *Grhl*-genes are also implicated in the aetiology of a number of human diseases, including cleft palate [27,28], spina bifida [5,14,29], cancer [30], ectodermal dysplasia [28] and asthma [31]. This incredible functional diversity is mediated, at least in part, through a *grh*/*Grhl* transcription factor binding to a promoter or enhancer element and regulating the subsequent transactivation or repression of target effector genes through recruitment of transcriptional co-factors. 

The *grh*/*Grhl* binding site is exceptionally well-conserved through evolution, broadly taking the form AA**C**CG**G**TT (with the first “C” and second “G” largely invariant in experimentally validated assays). Although several upstream regulatory factors, such as branchless/FGF8 and Erk [32,33], and interacting co-factors, such as LMO4 [10,34], have been described, the delineation of *grh*/*Grhl*-dependent transcriptional networks has largely focussed on the identification and characterisation of these direct downstream target genes. Many candidate genes have been experimentally validated in the literature, both through targeted approaches (e.g., *Tgm1*/5 [35], *ARHGEF19* [36], *Dsg1* [37], *eng2a* [16], *spec1* [16], *edn1* [17] and *PTEN* [30], and, more recently, through large-scale screening approaches, such as RNA-SEQ and ChIP-SEQ. However, the precise mechanisms by which the *grh/Grhl*-dependent transcriptome regulates numerous disparate cellular and developmental functional processes are still unclear. Nevertheless, it is clear that the functional interaction between *grainyhead*-signalling and target gene regulation is dependent on tissue, and developmental/disease stage and organism, and the incredible heterogeneity of these regulations suggest that delineation of these *grh*/*Grhl* transcriptional networks will shed significant light on both embryonic development and disease. 

Currently, 41 Microarray/RNA-SEQ datasets probing Grhl function have been reported in the literature, from both *Drosophila* and mouse tissues, as well as several human cancer/short-hairpin RNA (shRNA) cell lines (Table 1). In order to delineate the *grh*/*Grhl* transcriptional network, we have interrogated these datasets to bring greater clarity to the *Grainyhead*-dependent transcriptome, and to begin to understand the critical effector genes that operate broadly across disparate contexts, as well as identifying those that have more restricted roles in specific processes, particularly epithelial development.

Lastly, using our recently described *grhl3^-/-^* zebrafish that present with epithelial defects of the enveloping layer, EVL [7], we performed Q-RT-PCR to determine the expression of zebrafish orthologues of the ten most differentially regulated epithelial genes, thereby functionally testing the predictive strength of our meta-analysis approach in a novel animal model. Together, this study collates multiple “big-data” approaches and provides an experimental pipeline for determining and testing novel *grh/Grhl*-dependent functional interactions.

## 2. Methods

### 2.1. RNA-SEQ and Microarray Meta-Analysis and Database Construction 

The datasets from individual studies were converted to a common file format (.csv) and imported into MySQL (www.mysql.com), a relational database management system that enables the identification of relationships between datasets. Inside the database, the data were combined into species-specific tables. Using information from Ensembl v.91, gene symbols from the original human data files were mapped to the human Ensembl gene_stable_ids; gene symbols from other species were mapped to human gene symbols and gene_stable_ids of their orthologous human genes. After mapping, all the human-mapped data were joined together into a single table, searchable by the original gene symbol in every species, as well as the gene symbol and gene_stable_id of the human orthologue, publication name, dataset name, type of *Grhl* regulator, and direction of *Grhl* regulation (positive or negative), and were designated as being derived from either “cell-line” or “tissue”. For interactive visualization and exploring, the data were exported from the MySQL database, re-structured and uploaded into the Ordino tool (https://ordino.caleydoapp.org). To achieve a common gene ranking, the fold change rank-based scores from individual datasets were aggregated into the sum of scores for each gene, separately for positively and negatively regulated genes. For datasets where p-values/false discovery rates (FDRs) were available, another set of scores, based on p-value/FDR ranks, and computed independently for positively and negatively regulated genes, are also available. For singular genes, and the direction of regulation, *p*-values/FDRs from multiple datasets were combined into a single p-value, using the standard Fisher’s method. The ranks were then transformed into normalised scores that permitted aggregation of rank-based information from datasets comprising different numbers of genes. In order to do this, we used a scoring function f(r) =1/r^k, where k was an arbitrary parameter, allowing us to determine the significance of a genes’ rank across datasets. The constant of k = 3 was chosen, as it balanced the number of datasets that a given gene appeared in with instances in which this gene was highly ranked within an individual dataset. Therefore, the top ranked gene (Rank #1) within a given dataset would achieve a normalised score of 1 (1/1^3), whereas, if a gene did not appear within a particular dataset, it would be given a score of 0. 

### 2.2. Zebrafish Experimentation

All zebrafish experiments were conducted in accordance with La Trobe University guidelines for the care and housing of zebrafish, under Animal Ethics Committee approval number AEC16-91. The *grhl3^-/-^* fish line was reported previously [7].

### 2.3. RNA Extraction and cDNA Synthesis

RNA was isolated from *grhl3^-/-^* and wild type embryos (at 48 hours post-fertilisation; hpf) as described previously [38]. cDNA was synthesized (from three biological replicates, using WT and phenotypic *grhl3^-/-^* embryos at 48hpf, utilising 50 embryos per group per replicate) using the iScript™ cDNA (Life Science Research, Bio-Rad, Hercules, CA, USA) synthesis kit, according to the manufacturer’s instructions. Quantitative Reverse Transcription Polymerase Chain Reaction (Q-RT-PCR) using SsoFast™ EvaGreen® Supermix (Bio-Rad) and a BioRad CFX96 Real-Time System/C1000 Thermal Cycler was employed to quantify expression levels of 10 genes (comprising 17 fish orthologues) that were predicted to be differentially regulated in epithelial establishment following loss of *grhl3*, using methodology described previously [38]; expression levels were normalised to the EF1α housekeeping gene, as described previously [7]. Oligonucleotide sequences used for Q-RT-PCR are shown in Table S1. 

## 3. Results

### 3.1. Meta-Analysis Reveals the Most Consistently Differentially-Regulated Genes Following grh/Grhl Misexpression 

**Figure 1:**
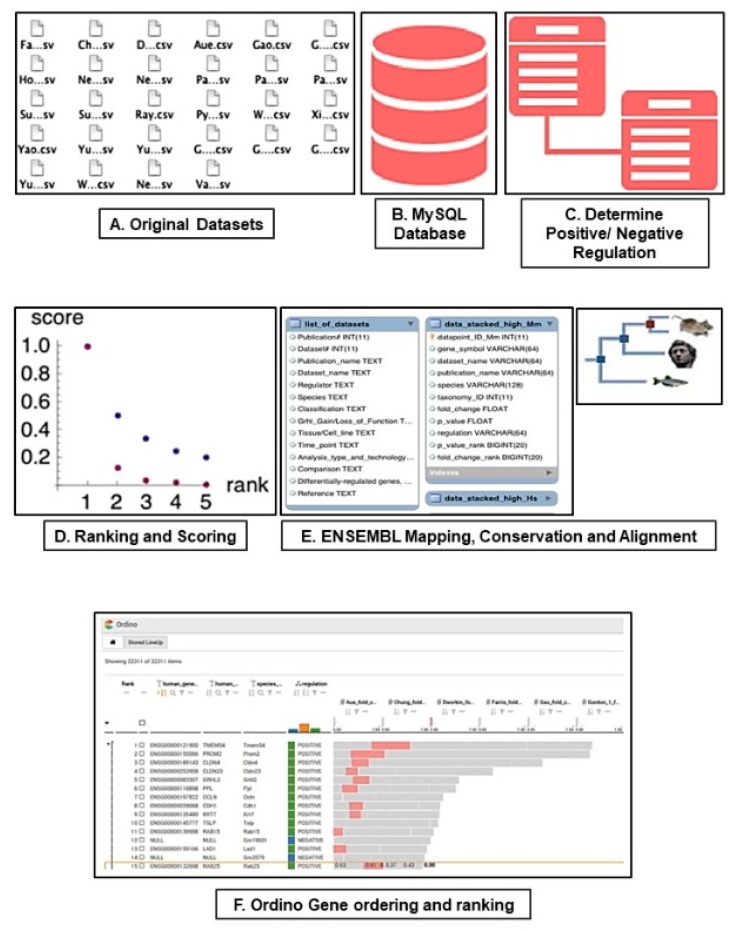
**Meta-analysis pipeline methodology** All published *grh/Grhl* Microarray/RNA-SEQ datasets were converted to a common file format (.csv, **A**) and imported into MySQL (**B**). Next, we determined whether genes within each dataset were positively or negatively regulated following functional modulation of *grh/Grhl* (**C**), and genes across the datasets were ranked according to their regulation within each dataset. To achieve a common gene ranking, the fold change rank-based scores from individual datasets were aggregated into the sum of scores for each gene, separately for positively and negatively regulated genes. (**D**). Each gene symbol within all datasets was mapped to the appropriate gene using Ensembl. After mapping, a table of all the human-mapped data was generated, searchable by the original gene symbol in every species (**E**). For interactive visualization and exploring, the data were exported from the MySQL database, re-structured and uploaded into Ordino (**F**).

In order to analyse the *grh*/*Grhl* transcriptome across multiple disparate biological contexts, we standardised all published *grh/Grhl* Microarray/RNA-SEQ datasets into a common format that allowed for direct comparison of differing methodologies and results, together with the corresponding published fold-changes and p-values (Figure 1). Differences in methodologies and data reporting in published studies created the need to integrate lists of regulated genes that were of different lengths, selected using different (study-specific) criteria. As studies varyingly examined the effects of either loss or gain of function of *grh*/*Grhl*, we divided the data based on whether a gene was positively or negatively regulated under normal homeostatic conditions. That is, if a gene was found to be downregulated in an experimental paradigm following loss of *grh/Grhl*, we deemed it to be positively regulated. Next, the positively and the negatively regulated genes were separately ranked, on the basis of the reported fold-change within each dataset (Figure 1).

**Figure 2:**
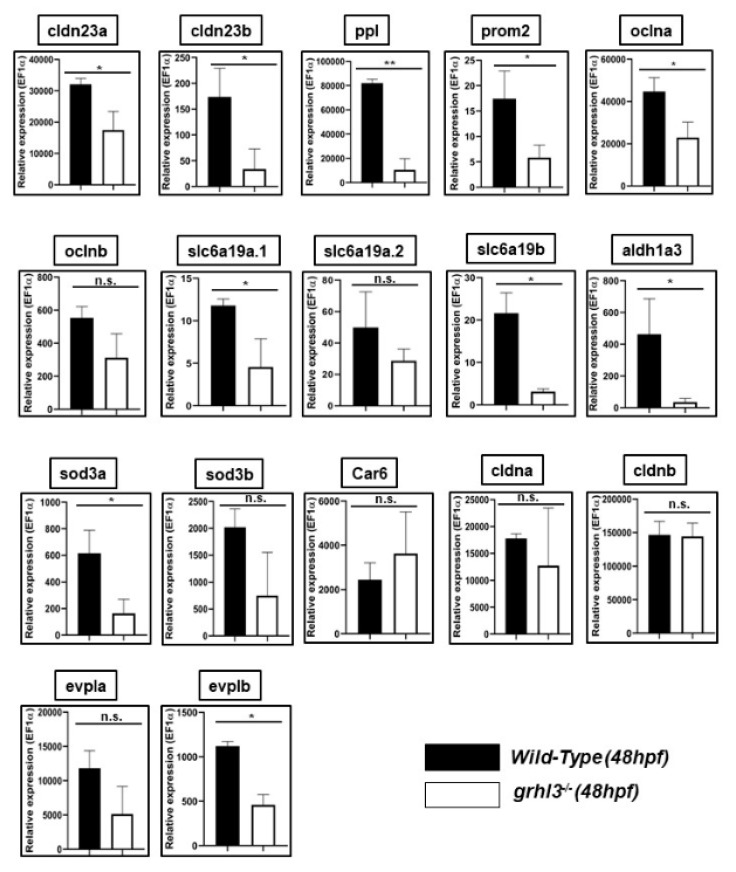
Q-RT-PCR analysis of zebrafish orthologue expression in 48 hours post-fertilisation (hpf) WT and *grhl3*^-/-^ embryos. Gene expression was normalised to expression of housekeeping gene EF1a. Of the 18 fish orthologues selected, 10 showed differential expression in WT (black bars) vs *grhl3^-/-^* (white bars) embryos. * *p* < 0.05, ** *p* < 0.01, n.s., non-significant.

Our analysis allowed us to determine the genes that are both most frequently and most substantially differentially regulated, following gain/loss of *grh/Grhl* function. From these comparisons, we determined which genes exhibited the greatest degree of regulation following disruption of grh/*Grhl* signalling. We determined this by: (1) frequency of altered expression (the number of datasets a given gene appeared in as “regulated”); and (2) degree of regulation, measured by the score, based on the rank given to a gene in a particular dataset, with respect to other differentially regulated genes. These analyses allowed us to reveal the genes that appeared within at least one dataset and determine the top differentially regulated genes following grh/Grhl de-regulation or misexpression, across all biological contexts thus far tested and described in the literature (Figure 2). 

From these analyses, we compiled a list of the top 50 most-highly differentially regulated genes (following *grh/Grhl* modulation) across various tissues and cell lines, and at different developmental time points (Table 2). As *grh/Grhl* transcription factors bind to the promoter of target genes in order to drive transcription, we determined whether any of these genes had previously been identified as being bound by *grh/Grhl* factors in ChIP or ChIP-SEQ datasets. In total, 15 of the top 50 genes in our list (30%: *tmem54, prom2, cldn4, ppl, cdh1, rab15, lad1, rab25, Epcam, Tacstd2, st14, Esrp1, Prss22, spint1* and *prss8*) are known to be bound to *grh/Grhl* factors, supporting the robustness of our analysis algorithm. Additionally, seven genes in our list—*cldn4, rab15, rab25, epcam, cdh1, tslp,* and *spint1—*had been previously characterised as direct *Grhl*-target genes through biological validation experiments, further highlighting the predictive strength of our approach.

### 3.2. Biological Pathways Regulated by grhl in Epithelia as Identified by GO Analysis 

In order to determine the overall biological processes regulated by the grh/*Grhl* pathway, we performed GO (Gene Ontology) pathway analysis, using gProfiler’s GOStat (Gene Group Functional Profiling), with the default background set of all human genes. Mindful of obvious limitations, such as tissue origin, disease state and temporal/developmental time point, we nonetheless analysed all the disparate datasets to determine an “overall” snapshot of *grhl*-target gene biology (Table 3). We found that this analysis largely confirmed previous known roles of the *Grhl* family. The major biological functions ascribed to genes regulated by the *Grhl* family were “cell–cell adhesion” ”tissue (epithelia/epidermis) development”, “animal organ/skin development”, “water homeostasis” and “epithelial cell morphogenesis”, consistent with known *grh/Grhl*-dependent functions in epithelial morphogenesis and maintenance, as well as preventing trans-epidermal water loss and maintaining epithelial integrity through suppressing the epithelial–mesenchymal transition (EMT). Somewhat surprisingly, the GO term “protein cross-linking” was also highlighted in our *grh/Grhl* transcriptome analysis. *grh/Grhl* factors are thought to be constitutively bound to the promoters/enhancers of target genes, achieving transcriptional specificity through the recruitment of partner protein co-activators as development proceeds [39]. These co-factors may include members of the Polycomb and Trithorax groups in Drosophila [39,40], and the vertebrate transcription factor LMO4 [34] in mice, although, as few such co-factors are known, enrichment of this GO term may suggest a novel, hitherto unsuspected *grh/Grhl* role in the assembly, formation or maintenance of the core transcriptional apparatus.

### 3.3. Grhl-Dependent Target Genes are Well-Conserved between Mouse and Human; Less so between Mouse and Drosophila

In order to determine how well conserved the *grh/Grhl*-dependent transcriptional pathways were between mouse, *Drosophila* and human, we generated separate lists for each organism, using only species-specific, large-scale microarray/RNA-SEQ datasets. This analysis comprised 20 datasets from mouse, six datasets from *Drosophila*, and 12 datasets from human (Table 1). Using the top 50-ranked mouse genes as our root list for comparison, we determined which of these genes were ranked in the top 10% differentially-regulated genes in both *Drosophila* (in the top 1300 of ~13,000 genes in the *Drosophila* genome) and human (top 2500 out of ~25,000 total human genes), reasoning that genes which fell below this ranking threshold were less likely to be biologically-relevant in the context of *grh/Grhl* transcription (Table 4). Our analyses showed that 32 of the top 50 mouse genes had an identifiable *Drosophila* orthologue, with 4/32 orthologues of these mouse genes—*Grhl2 (grh), Car6 (CAH7), Car2 (CAH1)* and *unc93a (GC4928)—*also being differentially-regulated in *Drosophila*. Unsurprisingly, the degree of conservation was greater in human, with 47/50 human orthologues identified, and 17/47 of these mouse targets appearing in the top 10% differentially regulated human genes. Moreover, 13 of these orthologues—*PROM2, CLDN4, PPL, GRHL2, CDH1, LAD1, RAB25, TMPRSS13, AP1M2, KLK6, SFN, CLDN1* and *RPTN*—appeared within the top 500 (top ~2%) most differentially regulated genes in humans, suggesting that these genes may substantially underpin *Grhl*-dependent transcriptional networks in mammalian species. Analysis of these 13 genes shows that eight of these—*Prom2, Cldn4, Ppl, Cdh1, Lad1, Tmprss13, Cldn1* and *RPTN*—are involved in maintaining structural integrity within epithelia, particularly the epidermis. Of the others, there is a member of the Ras superfamily of small GTPases, involved in vesicular trafficking to the cell membrane (*Rab25*), a transcription factor (*Grhl2* itself), a serine protease (*KLK6*), a clathrin-associated adaptor in the Golgi/endosome network (*Ap1M2*) and a cell-cycle checkpoint protein (SFN). This suggests that no other biological processes are as well conserved among Grhl-differentially regulated transcripts across multiple species as epithelial and epidermal integrity. These data are particularly striking, given the large differences in the source material used to generate each of the datasets, comprising different organisms, disease states, developmental timepoints and manipulating different *grh/grhl* orthologues.

### 3.4. Refining Meta-Analyses to Encompass Only Large-Scale Datasets Generated from Epithelial Tissue or Cancer Cell Lines Reveals grh/Grhl-dependent Transcriptome Specificity

As the *grh/Grhl* family are key regulators of epithelial establishment, development, maintenance and homeostasis, we refined our meta-analyses to only include datasets generated from primary epithelial tissues (Table 1). These data encompassed 18 datasets from seven separate publications, and restricted our data to *Grhl*-dependent transcriptomes, derived from epithelia from mouse embryonic skin [10], bladder [41], non-neural ectoderm [42] and lung epithelium [43], human neonatal epidermal keratinocytes [44], and undifferentiated primary human bronchial epithelial cells [31], as well as adult mouse and human psoriatic skin [45], and allowed us to identify the most-differentially regulated genes in epithelial development, the top 50 of which are shown in Table 5. This list includes numerous well characterized genes, known to be expressed in epithelia, which are involved in the maintenance of epithelial integrity and structural stability, such as *Claudins 1/4/23*, *Periplakin*, *Envoplakin*, *Occludin* and *Prominin 2* (Table 5). Interestingly, the top 50 genes include five “predicted” genes—*Gm19601*, *Gm3579*, *231000H18Rik*, *Gm20305* (possible pseudogene) and *Gm10639—*that are yet to be ascribed a function. Next, we determined which of these top 50 “epithelial” *grh/Grhl*-regulated genes may also be de-regulated in *Grhl*-influenced cancerous cells (Table 6). We generated a dataset of differentially regulated genes in those RNA-SEQ/Microarray datasets that were designated as being derived from “cancer cell-lines” (Table 6). These experiments typically included control cancer cells, compared to those in which *Grhl* activity was modulated through short-hairpin (shRNA) knockdown, and included 4T1 breast tumor cells [46], human mammary epithelial (HMLE-Twist-ER) cells [47], MSP (mesenchymal sub-population) cells obtained from HMLE cells [48], OVCA429 (ovarian cystadenocarcinoma; intermediate epithelial (IE) phenotype) cells [49] and LNCaP (human prostate carcinoma) cells [50]. Surprisingly, only two genes appeared in both the “epithelial targets” and “cancer targets” tables—*Tmem54* and *Claudin*-*4*, a previously experimentally validated *Grhl*-target. We did note several instances of different family members expressed across both sets (e.g., *Sod3, Krt6b/78, Tmprss4/11bnl* and *Cldn1/23/b8* in epithelia, and *Sod1/2, Krt7/19, Tmprss13* and *Cldn7* in cancer), suggesting that loss of *Grhl*-mediated transcription may lead to similar biological consequences, albeit mediated via different (possibly secondary, or indirect) target pathways. However, these results indicate that the *Grhl*-dependent transcriptome is largely context and tissue-type dependent, with little evidence of conserved mechanisms operating in epithelial homeostasis and epithelial cancer.

### 3.5. Expression of Differentially-Regulated Epithelial Genes in a Novel Vertebrate Model

In order to determine the predictive power of our meta-analysis approach, to identify novel *grhl*-dependent putative target genes (both direct and indirect), we analysed the expression of our top-ranked genes that were differentially regulated in epithelia, by performing Q-RT-PCR on cDNA from *grhl3^-/-^* mutant zebrafish. This model was utilised to test our meta-analyses, as none of the large-scale datasets were generated from zebrafish tissue, hence, this model would speak to the genetic conservation of *grhl*-dependent pathways across vertebrates. 

From the genes that were differentially regulated in epithelia (Table 5), we identified the top 10 genes with an identifiable zebrafish orthologue, or orthologues. The roles of these ten mammalian genes—*Cldn23, Ppl, Prom2, Ocln, Slc6a19, Aldh1a3, Sod3, Car6, Cldn4 and Evpl—*were sub-functionalised into a total of 17 zebrafish orthologues, based on sequence conservation homology (Table S2). Next, we performed Q-RT-PCR on fifty, 48 hours post-fertilisation (hpf) WT and *grhl3^-/-^* embryos (experiment performed in triplicate, see methods) to determine the relative expression of each of these fish orthologues. Of the 17 zebrafish orthologues analysed, we found that 10—*cldn23a, cldn23b, ppl, prom2, oclna, slc6a19a.1, slc6a19b, aldh1a3, sod3a* and *evplb—*showed statistically significant differences in expression between WT and *grhl3^-/-^* fish (primarily down-regulation). We utilised whole embryos for our analyses, not just epithelial tissue, and we only performed analyses at a single developmental timepoint. Nonetheless, even within these experimental constraints, the fact that 10/17 zebrafish orthologues were significantly differentially regulated highlights the remarkable conservation of *grhl*-dependent pathways across the evolutionary spectrum, and the strong predictive power of our approach for identifying novel, biologically relevant transcriptional networks downstream of g*rh/Grhl* function.

## 4. Discussion

In this study, we interrogated large-scale transcriptomic data to uncover novel genetic mechanisms that operate downstream of activation or repression by *grh/Grhl* genes. We collected all currently published large-scale RNA-SEQ and microarray datasets from multiple disparate animal models, developmental timepoints, tissues and cancer cell lines, to delineate conserved *grh/Grhl*-dependent genetic networks. Using a novel, ranking-based algorithm approach to draw synergies between disparate methodologies, we identified the top-differentially regulated genes following gain or loss of grh/Grhl function in all datasets, as well as refining our analyses to the top-differentially regulated genes in primary epithelia and in cancer. We also examined the conservation of mechanisms in mouse, *Drosophila* and human genes, and, lastly, we tested the predictive strength of our meta-analysis approach to determine which of the top-ranked genes were differentially regulated in a novel vertebrate grhl-loss-of-function model, the zebrafish. Taken together, our study brings cohesion and clarity to the existing “big-data” approaches to tackle grh/Grhl-dependent transcription, and opens up new avenues (and identifies novel putative targets) to further experimentally delineate *grh/Grhl*-dependent transcriptional genetic regulatory networks.

Across the 41 disparate Microarray/RNA-SEQ datasets, of the top 50 genes we identified, 15 had previous connections to *Grhl* transcription factors, either through ChIP/ChIP-SEQ experiments to identify regions of genome occupancy by *Grhl* proteins, or through targeted biological experiments to empirically determine novel functional relationships. The genes to which *Grhl*-factors bind, and drive/repress transcription can be considered true “direct” target genes. A number of our top 50 genes fall into this category, namely, *Cldn4, Cdh1 (E-Cadherin), Rab25, Epcam, Esrp1* and *Spint1,* with a further nine genes—*Tmem54, Prom2, Ppl, Lad1, Tacstd2, St14, Prss22,* and *Prss8—*showing instances of promoter-binding by *Grhl*-factors in ChIP-analyses, but currently lacking empirical experimental validation to determine true biological interactions. As such, these nine genes would appear to be the prime candidates for future studies into identifying novel *Grhl*-target genes through established analyses, such as in vitro luciferase activation assays, genetic complementarity in animal models (e.g., through generating *Grhl*/gene-of-interest doubly heterozygous mice) or models to rescue *Grhl* loss-of function phenotypes, as we have shown previously by rescuing aberrant developmental phenotypes in *grhl2b* or *grhl3*-deficient zebrafish embryos, with *eng2a* [16] and *edn1* [17] mRNA micro-injection, respectively.

The *Grhl*-family are well-known in both embryogenesis and cancer. Multiple studies have highlighted substantial developmental abnormalities in animal models lacking *Grhl* factors. These include numerous skin formation, maintenance and healing defects [2,11,28,36,51], impaired neural tube closure [5], facial and palatal clefting [19,52], regulation of skull bone apposition [18] and impaired formation of the lower jaw [17]. These latter (craniofacial) defects are interesting, owing to the fact that they are secondary consequences of disrupted epithelial formation—either the overlying cranial skin not allowing cranial bones to grow and expand [18], or through decreased endodermal *edn1*-signalling from the endodermal epithelium to cranial neural crest cells [17]. These studies parallel recent findings in human craniofacial disorders, namely Van der Woude Syndrome (VWS), a congenital syndrome characterised by palatal clefting. Disruption of the thin epithelial layer covering the developing palatal shelves—the periderm—leads to clefting in both mice and humans due to adhesions forming between the palatal shelves and tongue in utero [19]. Critically, VWS is also known to be caused by mutations in a critical *Grhl*-co-factor, IRF6 [53] in both mice and humans. These studies show that understanding the *Grhl*-dependent transcriptome is likely to uncover further novel biological pathways in the aetiology of craniofacial (and other epithelial) disorders caused by a primary epithelial defect.

In the context of cancer, *Grhl*-factors perform tumor-suppression roles in the epithelia of both the skin [30] and oesophagus [54], as well as maintaining epithelial identity through suppression of the epithelial–mesenchymal transition (EMT) in cancers of epithelial origin [47]. Strikingly, and somewhat surprisingly, our study found virtually no correlation between the top 50 differentially regulated genes in normal epithelia as compared to cancerous cells, with only two genes—*Tmem54* and *Claudin4—*appearing in both lists. This could suggest that, in general terms, the normal *grh/Grhl-*dependent transcriptional pathways, that operate in epithelial development, homeostasis and maintenance, are not the most highly dysregulated in epithelial cancers. Caveats exist in this interpretation, chief among them being the lack of a direct comparison between normal and cancerous epithelia derived from the same source, as well as the substantial differences that exist between cells and tissues of origin utilised for the individual RNA-SEQ/Microarray datasets. Additionally, the published microarray/RNA-SEQ datasets defining the role of Grhl in cancers follow the experimental paradigm of inhibiting or over-expressing Grhl-factors in already tumorigenic cell lines, which is a separate biological question to analysis of the transcriptome of cancers that arise directly due to aberrant *Grhl*-function. Transcriptomic analyses of tumors arising in Grhl3-deficient mice, relative to non-cancerous adjoining tissue, such as the skin and esophagus [30,54], would add important data points to our current meta-analyses.

Overall, this study demonstrates that meta-analysis of previously published RNA-Seq datasets is able to identify novel targets of *grhl* transcription. The depth and robustness of this approach is clearly demonstrated by the fact that many of the genes identified via this method have been previously validated in the literature. Moreover, the experimental validation of altered expression of our identified target genes, using a vertebrate model that was not included in any of the published data sets, highlights the power of this type of analysis to predict conserved gene targets across different model organisms. Our experimental paradigm naturally has certain caveats and limitations, which any analysis must keep in mind, such as the degree and direction of target regulation in fish compared to mammals, the analysis of gene expression over separate developmental and, perhaps, adult timepoints, and specific analyses through ISH/IHC of mRNA/protein distribution, specifically in epithelial tissues, e.g., developing EVL and the skin. Moreover, grh (and presumably also Grhl) proteins are known to be post-translationally modified through processes such as phosphorylation [32,55], which further confounds the precise nature of grh/Grhl-dependent regulation (or co-regulation) of transcriptional targets. Nonetheless our experimental approach in fish successfully identified 10 gene orthologues that are significantly differentially regulated, as predicted by the meta-analysis database construction algorithms we have developed and utilised here. Lastly, our analyses did not determine which of these genes may be true direct targets, based on promoter occupancy of the target gene promoter by grh/Grhl, although such direct binding and activation/repression approaches would be a logical extension of our meta-analyses. Thus, our database is an excellent resource for researchers in the field of grhl function, helping to identify and categorise novel molecular pathways underlying various developmental and disease processes.

## Figures and Tables

**Table 1:** Published datasets used for meta-analysis. Microarray and RNA-SEQ datasets including tissue of origin were classified as being derived from either “primary epithelia”, “cell line—cancer”, “cell line—non-cancer”, “other mammalian” or “other non-mammalian”, for subsequent analyses.

**Gene**	**Species**	**Dataset**	**Tissue/Cell Line**	**Classification/Origin**	**Reference**
*grh*	*Drosophila melanogaster*	1	2–3 hour whole embryos	Other - non-mammalian	Nevil et al., *Genetics*, 2017
		2	11–12 hour whole embryos	Other - non-mammalian	Nevil et al., *Genetics*, 2017
		3	15–16 hour whole embryos	Other - non-mammalian	Nevil et al., *Genetics*, 2017
		4	13–16 hour whole embryos	Other - non-mammalian	Yao et al., *Development*, 2017
		5	13–16 hour whole embryos	Other - non-mammalian	Yao et al., *Development*, 2017
		6	Stage 16–17 whole embryos	Other - non-mammalian	Yao et al., *Development*, 2017
*Grhl*	*Neurospora crassa*	7	48 hour aerial hyphae and conidia	Other - non-mammalian	Pare et al., *PLoS ONE*, 2012
*Grhl2*	*Mus musculus*	8	E9.5 Non-neural ectoderm	Primary Epithelia	Ray et al., *Development*,2016
		9	E16.5 Lung epithelium	Primary Epithelia	Kersbergen et al., *Dev. Biol*., 2018
		10	E16.5 Lung epithelium	Primary Epithelia	Kersbergen et al., *Dev. Biol*., 2018
		11	E9.5 Cranial tissue	Other - mammalian	Pyrgaki et al., *Dev. Biol*., 2011
		12	Extraembryonic trophectoderm-derived tissues at E7.5	Other - mammalian	Walentin et al., *Development*, 2015
		13	E9.5 Placenta	Other - mammalian	Walentin et al., *Development*, 2015
		14	E9.5 First pharyngeal arch (PA1)	Other - mammalian	Carpinelli et al., *submitted*, 2019
		15	Immortalised Mouse Lung Epithelial Cells (MLE15)	Cell line - Non-Cancer	Varma et al*., J. Biol. Chem*., 2012
		16	NIH3T3 fibroblast cells	Cell line - Non-Cancer	Werner et al., *J. Biol. Chem*., 2013
		17	IMCD-3 (inner medullary collecting duct) cell line	Cell line - Non-Cancer	Aue et al., *J AmSoc Nephrol*, 2015
*Grhl2*	*Canis familiaris*	18	Madin-Darby Canine Kidney (MDCK) cells	Cell line - Non-Cancer	Pifer et al., *Mol. Biol. Cell*, 2016
		19	Madin-Darby Canine Kidney (MDCK) cells	Cell line - Non-Cancer	Pifer et al., *Mol. Biol. Cell*, 2016
*Grhl2*	*Homo Sapiens*	20	"Early Passage" Primary Normal Human Epidermal Keratinocytes (NHEK)	Primary Epithelia	Chen et al., *Cell Death Dis*., 2012
		21	"Late passage" NHEK	Primary Epithelia	Chen et al., *Cell Death Dis*., 2012
		22	Undifferentiated primary human bronchial epithelial cells	Primary Epithelia	Gao et al., *PNAS,* 2014
		23	4T1 breast tumour cells	Cell line - Cancer	Xiang et al., *PLOS ONE,* 2012
		24	HMLE-Twist-ER cells	Cell line - Cancer	Cieply et al., *Cancer Res.*, 2012
		25	MSP (mesenchymal sub-population) cells obtained from HMLE cells	Cell line - Cancer	Farris et al., *Mol Cancer Res*. 2016
		26	OVCA429 (ovarian cystadenocarcinoma; intermediate epithelial [IE] phenotype)	Cell line - Cancer	Chung et al., *Scientific Reports*, 2016
		27	LNCaP (human prostate carcinoma cells)	Cell line - Cancer	Paltoglou et al., *Cancer Res*. 2017
**Gene**	**Species**	**Dataset**	**Tissue/Cell line**	**Classification/Origin**	**Reference**
*Grhl3*	*Mus musculus*	28	Embryonic day 18.5 (E18.5) embryo backskin	Primary Epithelia	Yu et al., *Dev. Biol*., 2006
		29	E14.5 Bladder	Primary Epithelia	Yu et al., *EMBO J*, 2009
		30	E16.5 Bladder	Primary Epithelia	Yu et al., *EMBO J*, 2009
		31	E18.5 Bladder	Primary Epithelia	Yu et al., *EMBO J*, 2009
		32	E14.5 Skin	Primary Epithelia	Gordon et al., J *Clin. Invest*., 2014
		33	E16.5 Skin	Primary Epithelia	Gordon et al., J *Clin. Invest*., 2014
		34	E18.5 Skin	Primary Epithelia	Gordon et al., J *Clin. Invest*., 2014
		35	Adult Skin	Primary Epithelia	Gordon et al., J *Clin. Invest*., 2014
		36	Adult Skin	Primary Epithelia	Gordon et al., J *Clin. Invest*., 2014
		37	Adult Skin	Primary Epithelia	Gordon et al., J *Clin. Invest*., 2014
*Grhl3*	*Homo sapiens*	38	NHEK (Neonatal Human Epidermal Keratinocytes) cells	Primary Epithelia	Hopkin et al., *PLoS Genet*. 2012
		39	NHEKs and adult psoriasis skin	Primary Epithelia	Gordon et al., J *Clin. Invest*., 2014
*Grhl3-Isoform 1*	*Homo Sapiens*	40	Human Embryonic Kidney neuroepithelial cell line (HEK-293)	Cell line - Non-Cancer	Haendeler et al., *Arter. Thromb. Vasc. Biol*., 2013
*Grhl3-Isoform 3*	*Homo Sapiens*	41	HEK-293	Cell line - Non-Cancer	Haendeler et al., *Arter. Thromb*. *Vasc. Biol*., 2013

**Table 2:** The top 50 most differentially regulated genes in all biological contexts following modulation of *grh/Grhl* function. The overall top 50 differentially regulated (both positively and negatively) genes of *grh*/*grhl* as identified by analysis of all published Microarray and RNA-SEQ datasets. 15 out of the 50 predicted target genes had previously been validated in literature through either large-scale ChIP-SEQ genome mining experiments, or through experimental validation (highlighted in red).

GRHL Targets					
Rank	Gene	Regulation	Normalised Score	Known relationships with Grhl Transcription Factors	
				*Locus bound by GRHL (ChIP and ChIP-SEQ)*	*Experimental validation*
1	**Tmem54**	POSITIVE	0.184	*Aue, et al. 2015,Walentin et al. 2015*	
2	**Prom2**	POSITIVE	0.182	*Aue, et al. 2015,Walentin et al. 2015*	
3	**Cldn4**	POSITIVE	0.148	*Chung, et al. 2016, Werth et al., 2010. Varma et al., 2012*	*Senga, et al. 2012, Varma et al. 2012, Tanimizu and Mitaka 2013, Aue et al. 2015*
4	**Cldn23**	POSITIVE	0.113		
5	**Grhl2**	POSITIVE	0.089		
6	**Ppl**	POSITIVE	0.0868	*Aue, Hinze et al. 2015*	
7	**Ocln**	POSITIVE	0.0777		
8	**Cdh1**	POSITIVE	0.0754	*Aue, et al. 2015, Chung, et al. 2016, Werth et al. 2010. Varma et al., 2012*	*Werth, et al. 2010. Pyrgaki, et al. 2011, Xiang, et al. 2012, Gao, et al. 2013, Tanimizu and Mitaka 2013, Chung, et al. 2016, Nishino, Takano et al. 2017, Pan, et al. 2017*
9	**Krt7**	POSITIVE	0.0752		
10	**Tslp**	POSITIVE	0.0741		
11	**Rab15**	POSITIVE	0.0708	*Walentin et al. 2015*	
12	**Gm19601**	NEGATIVE	0.069		
13	**Lad1**	POSITIVE	0.0661	*Walentin et al. 2015*	
14	**Gm3579**	NEGATIVE	0.065		
15	**Rab25**	POSITIVE	0.0631	*Aue, et al. 2015*	*Senga, et al. 2012*
16	**Epcam**	POSITIVE	0.0618	*Chung, et al. 2016*	*Xiang, et al. 2012*
17	**Snx31**	POSITIVE	0.0607		
18	**Slc6a19**	NEGATIVE	0.0579		
19	**Zasp66**	NEGATIVE	0.0578		
20	**Ap1m2**	POSITIVE	0.0576		
21	**Sfn**	POSITIVE	0.0565		
22	**IL36RN**	POSITIVE	0.0562		
23	**2310001H18Rik**	POSITIVE	0.0562		
24	**Il17re**	POSITIVE	0.0558		
25	**Macc1**	POSITIVE	0.0558		
26	**Evpl**	POSITIVE	0.055		
27	**Gm20305**	NEGATIVE	0.0547		
28	**Aldh1a3**	POSITIVE	0.0538		
29	**Tacstd2**	POSITIVE	0.0531	*Chung, et al. 2016*	
30	**Sprr1a**	POSITIVE	0.0528		
31	**St14**	POSITIVE	0.0525	*Chung, et al. 2016*	
32	**Rbbp8**	POSITIVE	0.0521		
33	**Tmprss11b**	POSITIVE	0.0519		
34	**Cldn7**	POSITIVE	0.0515		
35	**CG10345**	NEGATIVE	0.0501		
36	**Sod3**	NEGATIVE	0.05		
37	**CG12480**	NEGATIVE	0.0499		
38	**Esrp1**	POSITIVE	0.0499	*Chung, et al. 2016*	*Xiang, et al. 2012*
39	**Prss22**	POSITIVE	0.0497	*Aue, et al. 2015*	
40	**Cyp2b19**	NEGATIVE	0.0497		
41	**Car6**	NEGATIVE	0.0497		
42	**Car2**	POSITIVE	0.0495		
43	**Fmo2**	NEGATIVE	0.0493		
44	**Elovl1**	NEGATIVE	0.0492		
45	**Elovl7**	NEGATIVE	0.0492		
46	**Defb3**	POSITIVE	0.0488		
47	**Spint1**	POSITIVE	0.0481	*Chung, et al. 2016, Walentin et al. 2015*	*Walentin, et al. 2015, Matsushita, et al. 2018*
48	**Prss8**	POSITIVE	0.0481	*Chung, et al. 2016*	
49	**Cldn6**	POSITIVE	0.0481		
50	**Gsta2**	NEGATIVE	0.0479		

**Table 3:** Gene Ontology (GO) analysis of the most differentially regulated cellular processes in all biological contexts, following modulation of *grh/Grhl* function. Functional Gene Ontology (GO) annotations are over-represented among the 200 genes positively regulated by *grh/grhl,* with the highest evidence of regulation across all species (highest fold change score aggregated over all the datasets).

Source	Term Name	Term ID	n of Term Genes	n of Query Genes	n of Common Genes	Corrected *p* Value
	Gene Ontology (Biological Process)					
BP	biological adhesion	GO:0022610	1351	80	18	1.69 × 10^−2^
BP	cell adhesion	GO:007155	1343	80	18	1.56 × 10^−2^
BP	cell-cell adhesion	GO:0098609	776	79	14	6.29 × 10^−3^
BP	cell-cell adhesion via plasma membrane adhesion molecules	GO:0098742	245	79	10	1.43 × 10^−4^
BP	calcium-independent cell-cell adhesion via plasma membrane adhesion molecules	GO:0016338	23	79	6	9.48 × 10^−7^
BP	developmental process	GO:0032502	6212	78	45	3.91 × 10^−3^
BP	anatomical structure development	GO:0048856	7793	78	44	1.43 × 10^−3^
BP	tissue development	GO:0009888	1926	66	27	6.12 × 10^−8^
BP	epithelium development	GO:0060429	1218	77	26	8.22 × 10^−10^
BP	epidermis development	GO:0008544	459	77	19	2.62 × 10^−11^
BP	multicellular organismal process	GO:0032501	7414	74	48	4.19 × 10^−3^
BP	multicellular organism development	GO:0007275	5321	78	41	3.72 × 10^−3^
BP	system development	GO:0048731	4760	78	40	5.09 × 10^−4^
BP	animal organ development	GO:0048513	3428	77	35	2.65 × 10^−5^
BP	skin development	GO:0043588	412	77	21	1.18 × 10^−14^
BP	water homeostasis	GO:0030104	69	77	5	2.14 × 10^−2^
BP	epithelial cell differentiation	GO:0030855	759	77	19	1.83 × 10^−7^
BP	epithelial cell differentiation involved in embryonic placenta development	GO:0060671	3	25	2	1.39 × 10^−2^
BP	epidermal cell differentiation	GO:0009913	353	77	16	1.13 × 10^−9^
BP	keratinocyte differentiation	GO:0030216	299	77	16	8.72 × 10^−11^
BP	keratinization	GO:0031424	224	77	15	2.25 × 10^−11^
BP	cornification	GO:0070268	111	73	11	8.16 × 10^−10^
BP	multicellular organismal water homeostasis	GO:0050891	64	77	5	1.48 × 10^−2^
BP	regulation of water loss via skin	GO:0033561	22	77	5	5.79 × 10^−5^
BP	establishment of skin barrier	GO:0061436	20	77	5	3.43 × 10^−5^
BP	epithelial cell morphogenesis	GO:0003382	32	25	3	2.65 × 10^−2^
BP	embryonic placenta morphogenesis	GO:0060669	25	31	3	2.17 × 10^−2^
BP	labyrinthine layer morphogenesis	GO:0060713	21	31	3	1.26 × 10^−2^
BP	branching involved in labyrinthine layer morphogenesis	GO:0060670	12	31	3	2.11 × 10^−3^
BP	epithelial cell morphogenesis involved in placental branching	GO:0060672	3	25	2	1.39 × 10^−2^
BP	peptide cross-linking	GO:0018149	59	64	5	3.86 × 10^−3^

**Table 4:** Conservation of differentially regulated genes between mouse, *Drosophila* and human across all biological contexts, following modulation of *grh/Grhl* function.

Grhl Targets - Mus Musculus											
Rank	*Mus musculus* Gene Symbol	Regulation	Average Normalised Score	Significant Differential Regulation in *Drosophila*				Significant Differential Regulation in in *Homo sapiens*			
				Orthologue	Rank	Regulation	Average Normalised Score	Orthologue	Rank	Regulation	Average Normalised Score
1	Prom2	Positive	0.163	prominin-like				PROM2	361	Positive	0.0116
2	Tmem54	Positive	0.154	*No known orthologue*				TMEM54	1062	Positive	0.0178
3	Cldn4	Positive	0.116	*No known orthologue*				CLDN4	28	Positive	0.0264
4	Cldn23	Positive	0.106	*No known orthologue*				CLDN23			
5	Ppl	Positive	0.0812	shot				PPL	110	Positive	0.0187
6	Grhl2	Positive	0.0752	grh	577	Positive	0.0323	GRHL2	159	Positive	0.0165
7	Rab15	Positive	0.0663	RabX4				RAB15			
8	Ocln	Positive	0.0655	Su(Tpl)				OCLN	1141	Positive	0.00717
9	Gm19601	Negative	0.0645	*No known orthologue*				POLR2K *			
10	Tslp	Positive	0.064	*No known orthologue*				TSLP	1957	Positive	0.00534
11	Gm3579	Negative	0.0608	*No known orthologue*				*No known orthologue*			
12	Cdh1	Positive	0.0592	CadN2				CDH1	138	Positive	0.0177
13	Snx31	Positive	0.0568	CG5734				SNX31			
14	Slc6a19	Negative	0.0542	CG43066				SLC6A19			
15	2310001H18Rik	Positive	0.0526	*No known orthologue*				*No known orthologue*			
16	Il17re	Positive	0.0522	*No known orthologue*				IL17RE			
17	Evpl	Positive	0.0514	shot				EVPL	2179	Positive	0.00501
18	Gm20305	Negative	0.0512	*No known orthologue*				*No known orthologue*			
19	Lad1	Positive	0.0508	Nuak1				LAD1	412	Positive	0.0102
20	Aldh1a3	Positive	0.0503	CG31075				ALDH1A3	1528	Positive	0.00614
21	Sprr1a	Positive	0.0494	CG17377				SPR1A			
22	Rbbp8	Positive	0.0487	*No known orthologue*				RBBP8NL			
23	Tmprss11b	Positive	0.0485	CG11836				TMPRSS2			
24	Sod3	Negative	0.0468	CG5948				SOD3			
25	Cyp2b19	Negative	0.0465	Cyp18a1				CYP2B6			
26	Car6	Negative	0.0464	CAH7	582	Positive	0.0323	CA6			
27	Car2	Positive	0.0463	CAH1	590	Positive	0.0323	CA2			
28	Fmo2	Negative	0.0461	Fmo-2				FMO2			
29	Defb3	Positive	0.0456	*No known orthologue*				HBETAD3			
30	Rab25	Positive	0.0453	Rab11				RAB25	241	Positive	0.0137
31	Prss8	Positive	0.045	CG16749				PRSS8			
32	Cldn6	Positive	0.045	*No known orthologue*				CLDN6			
33	Gsta2	Negative	0.0448	GstS1				GSTA1/2/3/5			
34	Aox4	Negative	0.0446	ry				AOX1			
35	Tmprss13	Positive	0.0441	Sb				TMPRSS13	5	Positive	0.0346
36	Smpdl3b	Positive	0.0437	CG32052				SMPDL3B			
37	Ap1m2	Positive	0.0434	AP-1mu				AP1M2	464	Positive	0.0105
38	Pax8	Negative	0.0433	sv				PAX8			
39	Klk6	Positive	0.0433	CG12951				KLK6	176	Positive	0.0157
40	Il33	Positive	0.0425	*No known orthologue*				IL33			
41	Fabp5	Positive	0.0424	fabp				FABP5			
42	Sfn	Positive	0.0419	14-3-3zeta				SFN	418	Positive	0.0109
43	Cldn1	Positive	0.0417	*No known orthologue*				CLDN1	442	Negative	0.0108
44	Slpi	Positive	0.041	CG5639				SLPI			
45	Unc93a	Negative	0.0409	CG4928	1219	Negative	0.041	UNC93A			
46	Krt6b	Positive	0.0408	LamC				KRT6B			
47	Macc1	Positive	0.0399	*No known orthologue*				MACC1			
48	Rptn	Positive	0.0393	*No known orthologue*				RPTN	79	Positive	0.0203
49	Gsta1	Negative	0.039	*No known orthologue*				GSTA1			
50	9930013L23Rik	Positive	0.0387	*No known orthologue*				CEMIP			

**Table 5:** The top 50 most differentially regulated genes in primary epithelia following modulation of *grh/Grhl* function. Orthologues of the genes highlighted in red were screened and validated in subsequent Q-RT-PCR experiments in *grhl3^-/-^* zebrafish.

Rank	Gene	Regulation	Normalised Score
1	Cldn23	POSITIVE	0.0954
2	Ppl	POSITIVE	0.0693
3	Prom2	POSITIVE	0.0689
4	Gm19601	NEGATIVE	0.0689
5	Tslp	POSITIVE	0.0684
6	Gm3579	NEGATIVE	0.065
7	Ocln	POSITIVE	0.0633
8	Slc6a19	NEGATIVE	0.0579
9	2310001H18Rik	POSITIVE	0.0562
10	Gm20305	NEGATIVE	0.0547
11	Aldh1a3	POSITIVE	0.0538
12	Tmprss11bnl	POSITIVE	0.0519
13	Snx31	POSITIVE	0.0517
14	Sod3	NEGATIVE	0.05
15	Cyp2b19	NEGATIVE	0.0496
16	Car6	NEGATIVE	0.0496
17	Cldn4	POSITIVE	0.0494
18	Fmo2	NEGATIVE	0.0493
19	Defb3	POSITIVE	0.0488
20	Gsta2	NEGATIVE	0.0479
21	Aox4	NEGATIVE	0.0476
22	Evpl	POSITIVE	0.047
23	Klk6	POSITIVE	0.0462
24	Il33	POSITIVE	0.0455
25	Cldn1	POSITIVE	0.0446
26	Slpi	POSITIVE	0.106
27	Unc93a	NEGATIVE	0.106
28	Krt6b	POSITIVE	0.105
29	Sprr1a	POSITIVE	0.105
30	Rptn	POSITIVE	0.102
31	Gsta1	NEGATIVE	0.101
32	Cemip	POSITIVE	0.0999
33	Fabp7	POSITIVE	0.0991
34	Adh6a	NEGATIVE	0.0985
35	Wfdc12	POSITIVE	0.0961
36	Fxyd4	POSITIVE	0.096
37	Krt78	NEGATIVE	0.0956
38	Tmem54	POSITIVE	0.0953
39	Gldc	NEGATIVE	0.0951
40	Gdpd1	NEGATIVE	0.0945
41	Slurp1	POSITIVE	0.0939
42	Hyal4	NEGATIVE	0.0934
43	Lce3f	POSITIVE	0.0933
44	Unc93a2	NEGATIVE	0.0932
45	Sptlc3	POSITIVE	0.0926
46	Pglyrp1	NEGATIVE	0.0924
47	Gm10639	NEGATIVE	0.0915
48	Gdpd3	NEGATIVE	0.0912
49	Cldnb8	POSITIVE	0.0912
50	Tmprss4	POSITIVE	0.0894

**Table 6:** The top 50 most differentially regulated genes in cancer cell lines following modulation of *grh/Grhl* function.

Rank	Gene	Regulation	Normalised Score
1	ALOX15	POSITIVE	0.045
2	CCDC88A	NEGATIVE	0.0405
3	FAT4	NEGATIVE	0.0345
4	EPCAM	POSITIVE	0.0345
5	IL36RN	POSITIVE	0.0345
6	AHSG	NEGATIVE	0.0345
7	Krt7	POSITIVE	0.0345
8	Sele	NEGATIVE	0.0345
9	AIM1	POSITIVE	0.0285
10	CD24	POSITIVE	0.0277
11	ALOXE3	POSITIVE	0.0274
12	SOD1	NEGATIVE	0.0274
13	ITGB6	POSITIVE	0.0274
14	SERPINE1	NEGATIVE	0.0274
15	NOX5	POSITIVE	0.0274
16	ALB	NEGATIVE	0.0274
17	Epcam	POSITIVE	0.0274
18	Ptges	NEGATIVE	0.0274
19	KRT19	POSITIVE	0.0263
20	TYMS	NEGATIVE	0.0248
21	ALOX12P2	POSITIVE	0.0239
22	Cat	NEGATIVE	0.0239
23	FGF2	NEGATIVE	0.0239
24	CRISP3	POSITIVE	0.0239
25	SYN3	NEGATIVE	0.0239
26	Cldn4	POSITIVE	0.0239
27	Otop1	NEGATIVE	0.0239
28	SLC1A6	POSITIVE	0.0217
29	NQO1	NEGATIVE	0.0217
30	TMPRSS13	POSITIVE	0.0217
31	ATOH8	NEGATIVE	0.0217
32	Cldn7	POSITIVE	0.0217
33	Xdh	NEGATIVE	0.0217
34	NCF2 (p67-PHOX)	POSITIVE	0.0202
35	GLUD1	NEGATIVE	0.0202
36	ITGA2	POSITIVE	0.0202
37	SMARCA1	NEGATIVE	0.0202
38	ALDH3B2	POSITIVE	0.0202
39	BRSK1	NEGATIVE	0.0202
40	Lamc2	POSITIVE	0.0202
41	Olfr1372-ps1	NEGATIVE	0.0202
42	SOD2	POSITIVE	0.019
43	ITGB8	POSITIVE	0.019
44	CDH2	NEGATIVE	0.019
45	A2ML1	POSITIVE	0.019
46	TMEM45A	NEGATIVE	0.019
47	Tmem54	POSITIVE	0.019
48	Dnahc6	NEGATIVE	0.019
49	CDH1	POSITIVE	0.0189
50	GALNT3	POSITIVE	0.0188

## Supplementary Materials

The following are available online at https://www.mdpi.com/2073-4425/10/11/876/s1, **Table S1:** Oligonucleotide sequences used in the present study; **Table S2:** Zebrafish orthologues of differentially regulated mammalian genes. The top 10 differentially regulated genes following *Grhl*-dependent modulation in primary epithelia that had identifiable zebrafish orthologues, showing the function of each gene in mammals. Zebrafish orthologues of these ten mammalian genes comprise a total of 18 separate genes.
