# Supplementary material for: Meta-Analysis of Grainyhead-Like Dependent Transcriptional Networks: A Roadmap for Identifying Novel Conserved Genetic Pathways
Source: Genes (Basel). 2019 Oct 31;10(11):876. doi: 10.3390/genes10110876 (PMC6896185; doi:10.3390/genes10110876)
Supplement: Supplementary file 1 [file genes-10-00876-s001.pdf]

Table S1. Oligonucleotide sequences used in the present study.

Zebrafish gene	Forward primer	Reverse primer
cldn23a	CAACCAACAAGGCAACGACC	CAAGCCGAGAGCAGTTACGA
cldn23b	TTTCCCCACTCCCCAAATCG	CCAGATCGATTGACTCCCCG
ppl	CTGTGGCCAGTGCTGACA	TCCTGTACTCTCCTTCTTGAGC
prom2	AGGACCTTTGCTTGGTGGAT	GCATCAATCTTGGGTTTCGGC
Gm3579 (exd2)	CCACTGAGCCCATTCTAGCC	GACACACGAACTCTTTTTACCCA
oclna	GGTCTGCTGGCTGACTATCC	GACTGAATTACGGACGGGCA
oclnb	CTCTCAAGGCATCGGGGAAT	GGAGGATATTCATCTGTGTCAAGC
slc6a19a.1	TCAGCAGCTCAACATCAGCA	ATCTCTTGGGCAGCAGGTTC
slc6a19a.2	CATCGATTTACGCCGCCATC	CAGAAACGTCTGCATGTCGC
slc6a19b	CCTTGGCTTGTCCTCCATGT	GTCAAATAGGGCCAGCCAGT
aldh1a3	TTCCTGGATTTGGACCGACG	ATAGACTCGTGATGCAGCGG
sod3a	ACATTCTCCGAGTTCAGACGG	CCTCTTTGTATGTGAAGCGCC
sod3b	TGGCCCAAAGGAAAAGCTGA	GGATTCCAGTGACTGCCGAA
car6 (ca6)	GACCCTGTGGAACGACTACC	GCTTGCCTTGAACCGCTTTT
cldna	CGCTGTTGATTATTGGCGGG	CCCTTGGTTCCTCCAGATCG
cldnb	GGTGCCTCAGCTCTGTTGAT	AAGTTCTTTCCGGAGGGTGC
evlpa	CACTAAACCGCACTGACCCT	TTGGGCTGCAACTCTCTGTT
evlpb	AGACGTTCACTGAGTCGCTG	TTACCTTCTCACCGCGCTTT
ef1α	TTGGAAACGGATATGCTCCA	TCCTTACCTGAACGCCTGTCA

Table S2. Zebrafish orthologues of differentially regulated mammalian genes. The top 10 differentially regulated genes following *Grhl*-dependent modulation in primary epithelia that had identifiable zebrafish orthologues, showing the function of each gene in mammals. Zebrafish orthologues of these ten mammalian genes comprise a total of 18 separate genes.

Grhl targets - Primary epithelia				
**Rank**	**Gene**	**Gene name**	**Zebrafish orthologue(s)**	**Function**
1	**Cldn23**	Claudin 23	*cldn23a, cldn23b*	Integral membrane proteins and components of tight junctions
2	**Ppl**	Periplakin	*ppl*	Components of desmosomes and of the epidermal cornified envelope in keratinocytes
3	**Prom2**	Prominin 2	*prom2*	Membrane glycoproteins
4	**Ocln**	Occludin	*oclna, oclnb*	Integral membrane proteins and components of tight junction strands
5	**Slc6a19**	Solute Carrier 6 Family Member 19	*slc6a19a.1, scl6a19a.2, slc6a19b*	Mediates resorption of neutral amino acids across the apical membrane of renal and intestinal epithelia
6	**Aldh1a3**	Aldehyde Dehydrogenase 1 Family Member A3	*aldh1a3*	Catalyzes the formation of retinoic acid
7	**SOD3**	Superoxide Dismutase 3	*sod3a, sod3b*	Protection of brain, lungs and other tissues from oxidative stress
8	**Car6**	Carbonic Anhydrase 6	*car6*	Reversible hydratation of carbon dioxide
9	**Cldn4**	Claudin 4	*cldna, cldnb*	Integral membrane protein
10	**Evpl**	Envoplakin	*evpla, evplb*	Component of the cornified envelope of keratinocytes

1
